# Supplementary material for: Natural selection and repeated patterns of molecular evolution following allopatric divergence
Source: eLife. 2019 Aug 2;8:e45199. doi: 10.7554/eLife.45199 (PMC6744222; doi:10.7554/eLife.45199)
Supplement: Supplementary file 3. [file elife-45199-supp3.docx]

Number of POGs with *Ka/Ks* values distributed in different categories.

| **Genera** | ***Ka/Ks*>=2** | | **1.1<=*Ka/Ks*<2** | | **0.9<=*Ka/Ks*<1.1** | | **0.5<=*Ka/Ks*<0.9** | | **0.1<= *Ka/Ks*< 0.5** | | **0<= *Ka/Ks*<0.1** | |
| --- | --- | --- | --- | --- | --- | --- | --- | --- | --- | --- | --- | --- |
|  | **Number** | **%** | **Number** | **%** | **Number** | **%** | **Number** | **%** | **Number** | **%** | **Number** | **%** |
| *Acorus* | 102 | 1.4% | 169 | 2.3% | 113 | 1.6% | 703 | 9.7% | 4542 | 62.6% | 1630 | 22.5% |
| *Calycanthus* | 115 | 1.2% | 413 | 4.3% | 286 | 2.9% | 1559 | 16.0% | 5435 | 55.9% | 1908 | 19.6% |
| *Campsis* | 72 | 0.9% | 285 | 3.7% | 171 | 2.2% | 986 | 12.8% | 3945 | 51.0% | 2271 | 29.4% |
| *Convallaria* | 125 | 1.6% | 396 | 5.2% | 214 | 2.8% | 977 | 12.8% | 3142 | 41.2% | 2763 | 36.3% |
| *Cornus-1* | 123 | 1.2% | 350 | 3.3% | 231 | 2.2% | 1339 | 12.6% | 6074 | 57.2% | 2501 | 23.6% |
| *Cornus-2* | 116 | 1.1% | 406 | 4.0% | 245 | 2.4% | 1265 | 12.4% | 5035 | 49.4% | 3131 | 30.7% |
| *Cotinus* | 128 | 1.3% | 433 | 4.3% | 248 | 2.5% | 1233 | 12.3% | 4683 | 46.8% | 3282 | 32.8% |
| *Croomia* | 173 | 1.9% | 322 | 3.5% | 200 | 2.2% | 1194 | 13.1% | 5205 | 56.9% | 2054 | 22.5% |
| *Dysosma* | 111 | 1.2% | 396 | 4.2% | 229 | 2.4% | 1398 | 14.7% | 4896 | 51.4% | 2497 | 26.2% |
| *Gelsemium* | 67 | 0.6% | 261 | 2.5% | 215 | 2.0% | 1574 | 14.9% | 7219 | 68.4% | 1221 | 11.6% |
| *Hamamelis* | 115 | 1.1% | 375 | 3.6% | 268 | 2.6% | 1609 | 15.5% | 6379 | 61.5% | 1628 | 15.7% |
| *Liquidarnbar* | 126 | 1.2% | 345 | 3.2% | 240 | 2.2% | 1407 | 13.0% | 6259 | 58.0% | 2409 | 22.3% |
| *Liriodendron* | 78 | 1.0% | 288 | 3.8% | 170 | 2.2% | 843 | 11.1% | 3509 | 46.4% | 2673 | 35.4% |
| *Meehania* | 85 | 0.8% | 286 | 2.5% | 215 | 1.9% | 1163 | 10.3% | 6543 | 58.1% | 2967 | 26.4% |
| *Menispermum* | 112 | 1.4% | 331 | 4.1% | 195 | 2.4% | 1038 | 12.9% | 3656 | 45.5% | 2707 | 33.7% |
| *Nelumbo* | 184 | 2.0% | 616 | 6.7% | 336 | 3.7% | 1778 | 19.5% | 4561 | 49.9% | 1664 | 18.2% |
| *Penthorum* | 93 | 0.9% | 344 | 3.4% | 207 | 2.1% | 1224 | 12.2% | 5300 | 53.0% | 2829 | 28.3% |
| *Phryma* | 119 | 1.1% | 394 | 3.6% | 240 | 2.2% | 1298 | 12.0% | 5691 | 52.7% | 3061 | 28.3% |
| *Sassafras* | 160 | 1.6% | 461 | 4.6% | 259 | 2.6% | 1293 | 12.8% | 5197 | 51.4% | 2738 | 27.1% |
| *Saururus* | 104 | 1.3% | 247 | 3.0% | 150 | 1.8% | 934 | 11.5% | 4850 | 59.5% | 1870 | 22.9% |
