# Supplementary material for: Natural selection and repeated patterns of molecular evolution following allopatric divergence
Source: eLife. 2019 Aug 2;8:e45199. doi: 10.7554/eLife.45199 (PMC6744222; doi:10.7554/eLife.45199)
Supplement: Supplementary file 10. [file elife-45199-supp10.docx]

Correlation among Ks in peak, relative abundance of genes in different ranges of *Ka/Ks* ratios and divergence time.

|  | ***Ks* in peak** | **0<= *Ka/Ks*< 0.1** | **0.1<= *Ka/Ks*< 0.5** | ***Ka/Ks*>=1.1** | **Divergence time** |
| --- | --- | --- | --- | --- | --- |
| ***Ks* in peak** | 1 ^d^ |  |  |  |  |
| ***Ka/Ks* < 0.1^a^** | -0.793969679 | 1 |  |  |  |
| **0.1 <= *Ka/Ks*< 0.5^b^** | 0.932460671 | -0.855543617 | 1 |  |  |
| ***Ka/Ks* >= 1.1^c^** | -0.534352806 | 0.196788535 | -0.6466 | 1 |  |
| **Divergence time** | 0.803400127 | -0.566396489 | 0.696386 | -0.39397 | 1 |

Note: a, relative abundance of genes in the category of 0<= *Ka/Ks*< 0.1.

b, relative abundance of genes in the category of 0.1<= *Ka/Ks*< 0.5.

c, relative abundance of genes in the category of *Ka/Ks*>=1.1.

d, correlation coefficient calculated by CORREL function in Excel.
